# Recent advances in understanding and managing cholestasis

**DOI:** 10.12688/f1000research.8012.1

**Published:** 2016-04-19

**Authors:** Martin Wagner, Michael Trauner

**Affiliations:** 1 Division of Gastroenterology and Hepatology, Department of Internal Medicine, Medical University of Graz, Graz, Austria; 2 Division of Gastroenterology and Hepatology, Department of Medicine III, Medical University of Vienna, Wien, Austria

**Keywords:** cholestasis, liver, hepatic, bile acid

## Abstract

Cholestatic liver diseases are hereditary or acquired disorders with impaired hepatic excretion and enterohepatic circulation of bile acids and other cholephiles. The distinct pathological mechanisms, particularly for the acquired forms of cholestasis, are not fully revealed, but advances in the understanding of the molecular mechanisms and identification of key regulatory mechanisms of the enterohepatic circulation of bile acids have unraveled common and central mechanisms, which can be pharmacologically targeted. This overview focuses on the central roles of farnesoid X receptor, fibroblast growth factor 19, and apical sodium-dependent bile acid transporter for the enterohepatic circulation of bile acids and their potential as new drug targets for the treatment of cholestatic liver disease.

## Introduction

**Figure 1.**
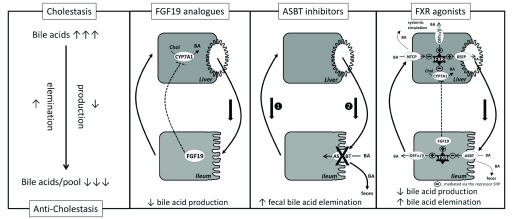
Principle anticholestatic mechanism of fibroblast growth factor 19 (FGF19) analogues, apical sodium-dependent bile acid transporter (ASBT) inhibitors, and farnesoid X receptor (FXR) agonists. Cholestasis results in the accumulation of bile acids in the enterohepatic bile acid circulation. Novel promising anticholestatic strategies aim to eliminate bile acids and reduce bile acid pool size predominately by either reducing *de novo* bile acid production or eliminating bile acids by interrupting enterohepatic bile acid circulation. Intrahepatic bile acid levels decrease. *Left panel:* FGF19 analogues mimic the action of endogenous FGF19, which is synthesized in the terminal ileum. FGF19 robustly represses hepatic *de novo* bile acid synthesis by blocking the rate-limiting enzyme of bile acid generation, cholesterol 7-alpha hydroxylase (CYP7A1). This reduces bile acid pool size and the amount of bile acids by suppression of the biliary loop of enterohepatic circulation. *Middle panel:* ASBT inhibitors selectively block bile acid re-uptake in the terminal ileum by blocking the bile acid transporter ASBT. Bile acids spill over into the colon and are lost via feces. This reduces bile acid pool size and the amount of bile acids by initially (1) suppression of the portal loop of enterohepatic circulation. *Right panel:* FXR agonists are not tissue specific but predominately activate FXR in the ileum and liver. FXR agonists suppress (-) bile acid synthesis via induction of FGF19-mediated CYP7A1 suppression from the ileum and via FXR- short heterodimer partner 1 (SHP)-mediated CYP7A1 repression from the liver. This reduces bile acid pool size. In addition, FXR agonists limit cellular bile acid accumulation by blocking ileal (via ASBT) and hepatic (via sodium taurocholate cotransporting polypeptide [NTCP]) bile acid uptake and by enforcing (+) ileal and hepatic (both via organic solute transporter α/β [OSTα/β]) bile acid export, leading to bile acid spill over into feces and systemic circulation.

Cholestasis is a hereditary or acquired impairment of bile formation and flow on either a hepatocellular or a cholangiocellular level, resulting in interruption of the enterohepatic circulation of bile acids. Classical hereditary defects comprise mainly mutations of transporter genes involved in hepatocellular bile formation such as *ATP8B1, BSEP*, and multidrug resistance protein 3 ( *MDR3*) underlying the classical progressive familial intrahepatic cholestasis type I-III, respectively ^1^, and rarely mutations of bile acid synthesis enzymes ^2^ or mutations in the bile acid receptor farnesoid X receptor (FXR) ^3^. In addition, mutations in the Notch signaling pathway frequently cause infant cholestasis in Alagille syndrome ^1^ and mutations/polymorphisms in *MDR3*, multidrug resistance-associated protein 2 ( *MRP2*), and *FXR* have been associated with intrahepatic cholestasis of pregnancy or drug-induced liver injury ^1^. Notably, mutations of sodium taurocholate cotransporting polypeptide ( *NTCP*) result in pronounced hypercholanemia without classical clinical features of cholestasis or impaired enterohepatic circulation ^4^. At the cholangiocyte level, hereditary defects of cystic fibrosis transmembrane conductance regulator ( *CFTR*) are the most common genetic contributors to cholestasis ^1^ and polymorphisms in anion exchanger 2 ( *AE2*) have been found as disease modifiers in primary biliary cholangitis (PBC) ^5^. In addition, mutations in tight junction proteins along the canalicular membrane of hepatocytes and cholangiocytes result in hereditary forms of cholestasis ^6^. Acquired forms of cholestasis originate from the hepatocellular level (e.g. estrogen-induced bland cholestasis, sepsis-induced cholestasis, and drug-induced cholestasis) or cholangiocyte or bile duct levels (e.g. PBC, primary sclerosing cholangitis [PSC], secondary sclerosing cholangitis, and intraluminal/extraluminal bile duct obstructions) or represent a mixture of hepatocellular/cholangiocellular origins ^7^. The common hallmark of various forms of cholestasis is impaired proper circulation of bile acids along the enterohepatic circulation resulting in the accumulation of potential toxic bile acids in the systemic circulation and intracellularly. A general goal in treating cholestasis, therefore, is to reduce hepatic and systemic bile acid accumulation and to decrease bile acid pool size ( Figure 1).

## Enterohepatic circulation of bile acids

Bile acids are formed in hepatocytes from hydroxylation of cholesterol by cholesterol 7-alpha hydroxylase (CYP7A1) or alternatively by CYP27A1 ^8^. The resulting primary bile acid chenodeoxycholic acid (CDCA) can be further hydroxylated to cholic acid (CA) by CYP8B1. Bile acids are exported across the canalicular membrane of hepatocytes into the bile duct lumen via distinct bile acid transporters, of which BSEP (ABCB11) transports the bulk of bile acids, accompanied by MRP2, the latter one transporting conjugated bilirubin and other xenobiotics. In addition, formation of primary bile requires active transport of phospholipids (via MDR3), cholesterol (via ABCG5/8), glutathione (also via MRP2), bicarbonate (via CFTR), and passive dilution by water ^9, 10^. Along the bile ducts, bile is further modified by bicarbonate-enriching mechanisms ^11, 12^ and further bile acid uptake mechanisms within the liver ^9, 11^. In the terminal ileum, bile acids are efficiently shuttled across enterocytes back into the portal circulation by active uptake into enterocytes via apical sodium-dependent bile acid transporter (ASBT) (SLC10A2) and exported via organic solute transporter α/β (OSTα/β) (SLC51) ^9, 10^. Only a few bile acids escape this high-capacity re-uptake and conservation mechanism by ASBT and spill over into the colon, where they are secondarily transformed by bacteria into deoxycholic acid (DCA) and lithocholic acid (LCA), taken back up by colonic diffusion, or excreted via the feces ^9, 10^. From the portal circulation, bile acids are selectively imported into hepatocytes by an active transporting mechanism, mainly consisting of NTCP (SLC10A1) and to a lesser extent organic anion-transporting polypeptide (OATP1B1). Bile acids that escape the hepatocellular import are spilled over into the systemic circulation and may eventually be eliminated via the kidney and urine ^9^.

Bile acid concentrations (and indirectly also composition via different sensitivities of bile acid sensors to various bile acid species) along the enterohepatic circulation are sensed at “checkpoints” in hepatocytes and enterocytes. Depending on the actual bile acid load in the enterohepatic circulation, further bile acids can be produced and more efficiently conserved or production can be repressed and bile acid excretion favored. In cholestasis, when bile acids accumulate, (hepato)cellular export and re-routing bile acids to renal excretion comprises an important adaptive system to reduce further potential toxic bile acid accumulation and cell damage ^9, 13^. The alternative export of bile acids is canalized by active bile acid transporter systems such as OSTα/β, MRP3, and MRP4 at basolateral membranes, which transport bile acids out of hepatocytes. In line with enforcing cellular export, import of bile acids into hepatocytes (via NTCP) and enterocytes (via ASBT) is being reduced ^9, 13^. Accumulating bile acids in hepatocytes also limit further bile acid production via repressing CYP7A1 at the transcriptional level. However, this very efficient mechanism to reduce bile acid generation in the enterohepatic circulation may be compromised in settings when bile flow is significantly impaired and fewer bile acids are sensed in enterocytes ^14, 15^. Fibroblast growth factor 19 (FGF19) is an ileum-specific enteric hormone released in proportion to bile acid concentrations in enterocytes and the most efficient repressor of CYP7A1 and hepatocellular bile acid synthesis ^14^. Bile acids induce FGF19 in the terminal ileum, which is released in the portal circulation and reduces bile acid synthesis in hepatocytes in a negative feedback fashion. In obstructive cholestasis, when bile flow is reduced and fewer bile acids reach the ileum, FGF19 levels decrease and hepatocellular bile acid production is augmented. This results in a paradoxical metabolic situation with further increase of bile acid synthesis despite hepatic accumulation of bile acids.

## Bile acid receptor FXR

The main sensor of bile acids in the enterohepatic circulation is the bile acid receptor FXR (NR1H4), which is ligand activated in the order of potency by CDCA > DCA > LCA > CA ^16^. FXR is a nuclear hormone receptor and transcription factor that heterodimerizes with the retinoid X receptor α (RXRα, NR2B1) and regulates the expression of genes involved in bile acid metabolism but also genes regulating glucose and lipid metabolism and inflammation ^17^. FXR can either directly induce or reduce gene transcription or indirectly repress genes via the common repressor short heterodimer partner 1 (SHP) (NR0B2), which is a direct positive target of FXR ^18^. FXR is highly expressed along the gastrointestinal system, liver, kidney, and to minor extents the adrenal glands ^19^. Generally, FXR activation reduces intracellular bile acid load in target tissues by repressing bile acid import transporters (i.e. *NTCP* and *ASBT*) and inducing bile acid export pumps (i.e. *BSEP*, *MRP2*, and *OSTα/β*) along with suppression of bile acid synthesis (i.e. *CYP7A1*) ^10, 17^. FXR regulates bile acid synthesis from the intestine via induction of FGF19 and in hepatocytes via SHP-induced repression of *CYP7A1* ^14^. For proper CYP7A1 repression by intestinal FGF19, sufficient hepatic SHP expression is required ^14^. In addition, FXR activation favors bile acid detoxification via induction of cytochrome p450 3A4 ( *CYP3A4*), sulfotransferase 2A1 ( *SULT2A1*), and UDP glucuronosyltransferase 2 family, polypeptide B4 ( *UGT2B4*) ^17^ and stimulates biliary phospholipid excretion via *MDR3* ( *ABCB4*), thereby also counteracting cholesterol gallstone formation ^20^. Besides its role for bile acid metabolism, FXR activation also shows anti-inflammatory properties by blocking NFκB-mediated inflammatory gene expression and immunomodulatory effects by facilitating homing and function of myeloid-derived suppressor cells, which function as a critical negative feedback loop in immune-mediated liver injury ^21– 23^.

## New concepts in treating cholestasis

### Concepts to induce choleresis

Currently, the only approved drug for treating chronic cholestatic disorders is the hydrophilic bile acid ursodeoxycholic acid (UDCA) ^24^. UDCA’s anti-cholestatic properties are mainly attributed to its choleretic effects by stimulating hepatocellular secretion of bile acids and organic anions post-translationally and by inducing/stabilizing a bicarbonate-rich protection “umbrella” along the biliary tree ^12^. Anti-apoptotic and anti-inflammatory actions may additionally support UDCA’s beneficial anticholestatic action. Its clinical efficacy is limited because only approximately two-thirds of patients with PBC respond to UDCA therapy ^25^ and in PSC patients UDCA has no effect on transplant-free survival ^26^.

NorUDCA is a side chain shortened UDCA derivative, which induces bicarbonate-rich hypercholeresis as a result of cholehepatic shunting of conjugation-resistant NorUDCA and shows additional anti-inflammatory and anti-fibrotic qualities ^27– 30^. In contrast to UDCA, NorUDCA improved sclerosing cholangitis in Mdr2 knockout mice as a model system for PSC while UDCA even aggravates cholestatic liver injury in these animal models ^28, 29, 31^. Recently, a phase II clinical trial with NorUDCA for PSC has been completed and the full data are eagerly awaited ^32^. Both UDCA and NorUDCA, to a large extent, counteract cholestasis by their choleretic effects targeting impaired bile flow. 

### Concepts to reduce bile acid pool size

While UDCA and NorUDCA counteract cholestasis and impaired bile flow by primarily inducing bile-acid independent choleresis and modifying bile acid pool toxicity, another line of currently developed anticholestatic strategies targets enterohepatic circulation to primarily reduce bile acid pools. FXR agonists, FGF19 mimetics, and ASBT inhibitors with clearly defined modes of action are the most promising representatives and are currently being tested in phase II and phase III clinical trials.

***FXR agonists.*** FXR represents the central integrator of bile acid homeostasis and, once activated, results in the reduction of cellular bile acid levels. Although FXR activation by endogenous bile acid accumulation is intended to counteract potential toxic bile acid levels, its endogenous activation in chronic cholestatic liver diseases is apparently too weak for disease self-limitation. Synthetic and semi-synthetic FXR agonists, with higher affinity and potency to activate FXR, have therefore been successfully tested in animal models of cholestasis. In LCA- and ethinyl estradiol-induced cholestatic rats, the semi-synthetic steroidal FXR ligand obeticholic acid (OCA, formerly also referred to as 6-ethylchenodeoxycholic acid [6-ECDCA]), which is currently being tested in several clinical phase II and III studies for PBC and PSC, was able to restore reduced bile flow and improve cholestasis in several preclinical animal models of cholestasis ^33, 34^. Interestingly, in a mouse model of cholestasis resembling PSC (i.e. Mdr2 knockout mice), OCA did not show beneficial anticholestatic effects in this model, although ileal FGF15 was induced and hepatic Cyp7a1 repressed. Only the even more potent FXR-activating capacity of the steroidal dual FXR/G-protein-coupled bile acid receptor 1 (TGR5) agonist INT-767 improved cholestasis along with robustly induced bicarbonate-rich choleresis and reduction of biliary bile acid output ^35^. It is likely that species differences and differences in the cholestatic models (i.e. complete absence of biliary phospholipids in the Mdr2 model) may explain these discrepancies. Non-steroidal FXR agonists (i.e. GW4064), which are also being investigated in clinical settings, improved markers of cholestasis as well as reduced hepatic bile acid accumulation in bile duct-ligated and α-naphthyl isothiocyanate-treated rats too ^36^. It is important to note that steroidal FXR agonists (e.g. OCA, CDCA, and INT-767) activate FXR in hepatocytes and enterocytes as well and therefore beneficial effects of FXR agonism may be explained by concerted action of both hepatic and enteric FXR stimulation. Thus, beneficial effects of steroidal FXR agonism likely result from reduction of bile acid pool size along with stimulation of (bile acid-independent) bile flow. Non-steroidal FXR agonists, such as fexaramine, GW4064, PX-102, or various derivatives, are currently being developed and tested by different companies and may have different tissue selectivity and metabolic effects. Interestingly, when FXR was selectively overexpressed in the intestine of various mouse models of intrahepatic and extrahepatic cholestasis (i.e. bile duct ligation, α-naphthyl isothiocyanate treatment, and Mdr2 knockout mice), bile acid pool size was substantially reduced and cholestasis improved in these models ^37^. Similarly, the gut-restricted non-steroidal FXR agonist fexaramine robustly induces intestinal FGF15 without any hepatic FXR agonistic effects and significantly reduces serum bile acid levels, at least in a model of diet-induced obesity ^38^. This suggests that potentially ileal FXR stimulation alone may be sufficient to counteract cholestasis. However, comparable experiments, where only hepatic FXR is activated, have not been performed to further dissect ileal and hepatic requirements of anticholestatic effects. Therefore, from animal experiments, it is not entirely conclusive which FXR, ileal or hepatocyte or both, is required to target and if the effects of FXR activation are more dependent on stimulation/restoration of (bile acid-independent) bile flow or repression of bile acid synthesis and pool size or both.

In a human clinical phase II trial with PBC patients, OCA treatment showed significant improvement of alkaline phosphatase (AP) as the main readout marker of cholestasis ^39^. Clinically, the major side effect was dose-dependent pruritus, and biochemically an unfavorable trend in the cholesterol profile with decreased high-density lipoprotein (HDL) cholesterol was observed ^39^. The impact of OCA on cholesterol metabolism was even more pronounced in another clinical phase II trial in obese patients with non-alcoholic fatty liver disease (NAFLD) as the disease target, where not only HDL cholesterol decreased but also LDL cholesterol increased ^40^. Generally, the atherogenic lipid profile of OCA is less a concern in PBC patients, while it requires further evaluation in NAFLD patients with increased cardiovascular risk. It is important to note that in the PBC study, participants comprised only patients who did not respond adequately to their standard of care treatment with UDCA and thus were expected to progress with cholestatic liver disease over time. OCA improved laboratory-based clinical scoring parameters in a significant portion of patients to levels associated with normalization of prognosis ^39^. However, in total only 7% of patients completely normalized their AP levels, which might alternatively be explained by direct FXR-induced AP transcription rather than disease-related AP origin ^40, 41^. Also, the study’s duration was only 3 months and biopsies for histological correlation were not taken. From a mechanistic point of view, OCA treatment increased FGF19 serum levels and decreased 4-cholesten-3-one (C4) bile acid precursors and endogenous BA plasma levels ^39^, underscoring the ability of FXR agonists to reduce bile acid pool size. Apparently, data on bile flow, choleresis, and bicarbonate-rich flow were not determinable in human clinical trials. The beneficial effects of OCA in PBC patients are confirmed in larger long-term studies over 12 months, including a long-term extension study ^42, 43^. Currently, further trials in PBC and PSC are underway to study the long-term effects of OCA and more clearly evaluate OCA’s effects on lipid profiles.

***FGF19 mimetics.*** FGF19 is an endocrine hormone predominantly produced in the ileum, which very efficiently suppresses hepatic bile acid synthesis ^14^. In contrast to rodents, human FGF19 is also expressed in liver tissue and gallbladder epithelium under cholestatic conditions and positively correlates with disease severity ^44, 45^. It is assumed that hepatic and biliary FGF19 supports endogenous bile acid suppression in cholestasis via autocrine and paracrine mechanisms ^45^. Part of the beneficial effects of FXR agonists in cholestasis may be attributed to FXR-dependent induction of FGF19, and selective activation of FXR in the intestine even suggests that induction of ileal FGF19 may sufficiently treat cholestasis ^37^. This has led to trials explicitly testing FGF19 in cholestatic models. The potential tumorigenic effects of endogenous FGF19 have been overcome by novel engineered FGF19 mimetics which lack the proliferative potency of their endogenous mother compounds ^46– 48^. In bile duct-ligated and α-naphthyl isothiocyanate-treated mouse models of cholestasis, endogenous as well as non-tumorigenic FGF19 mimetics significantly suppress Cyp7a1 and total bile acid pools, resulting in markedly reduced liver injury ^46^. Similar effects were achieved in the Mdr2 knockout mouse model where FGF19 mimetics reversed fully developed liver injury, biliary fibrosis, and even cholecystolithiasis ^49^. In a phase I trial in human volunteers, FGF19 mimetics resulted in a 95% reduction of C4 bile acid precursor levels indicative of robust suppression of endogenous bile acid synthesis without showing apparent side effects ^46^. In a very recent phase II clinical trial in PBC patients unresponsive to UDCA treatment, FGF19 mimetics also robustly decreased C4 and slightly decreased total bile acid levels along with showing a significant reduction of AP levels. Main side effects, which overall were mild, included diarrhea, headache, and nausea ^50^. Besides its effects on bile acid metabolism, FGF19 has major metabolic effects on carbohydrate and lipid metabolism ^51^ and is therefore also regarded as a pharmacological approach to treat the metabolic syndrome and primary bile acid diarrhea ^52– 55^.

***ASBT inhibitors.*** ASBT maintains the enterohepatic circulation of bile acids by efficiently taking up 95% of bile acids from the intestine and preventing their fecal loss. ASBT knockout mice have a 20- to 30-fold increased fecal bile acid loss, which cannot be compensated by increased bile acid synthesis. These mice, therefore, have robustly reduced bile acid pool sizes by 80% despite significantly repressed FGF19 and increased Cyp7a1 activity ^56, 57^. Spillover of bile acids into the colon may cause bile acid-induced diarrhea, an effect which can be utilized in treating constipation but may also have malignant potential for colorectal cancer development ^58, 59^. Since ASBT knockout mice exhibit an increased cholesterol turnover, blocking ASBT also has a major impact on lipid metabolism and metabolic disorders ^57^. In the cholestatic Mdr2 knockout mouse model, ASBT inhibitors effectively decrease bile acid pool size, biliary bile acid concentrations, and bile flow, which results in significant improvement of liver injury and biliary fibrosis ^60, 61^. In a human phase I trial with healthy volunteers, ASBT inhibitors reduced total serum bile acids by almost 50% along with increased fecal bile acid excretion. FGF19 was decreased and C4 bile acid precursor levels increased but could not compensate for fecal bile acid loss ^62^. Conceptually, comparable effects on bile acid metabolism would be expected by treatment with (unspecific) bile acid sequestrants such as cholestyramine or colesevelam. However, side effects such as bloating, constipation, and sequestering of lipophilic vitamins limit their application ^63^. Interestingly, resin-bound bile acids appear to activate colonic TGR5 ^64^, while unbound colonic bile acids spilled over by ASBT inhibitors did not induce TGR5 signaling ^61^. Besides direct effects on bile acid metabolism, ASBT-induced spillover of bile acids into the colon may significantly affect the gut microbiome ^65, 66^ with potential secondary effects on cholestatic liver disease. These effects would be expected to be less apparent with resin-bound bile acids. Future clinical trials with ASBT inhibitors in patients with cholestasis are currently underway.

***What else is in the pipeline?*** Several other molecular targets with more or less well-defined modes of action and anticholestatic properties are currently being investigated. Among the most promising pharmacological options are PPARα ligands, which have shown clinical improvements in PBC patients in small clinical trials and await confirmation in larger multicenter trials ^24^. Mechanistically, PPARα ligands (i.e. fibrates) increase MDR3 expression and insertion into the canalicular membrane of hepatocytes and thereby stimulate biliary phospholipid secretion, rendering bile less aggressive ^67– 69^. This bile duct protective effect is further supported by reduction of bile acid synthesis (via CYP7A1 and CYP27A1), induction of bile acid detoxification (via CYP3A4) ^67^, and anti-inflammatory properties ^70^. Also, the glucocorticoid receptor is a putative target in the treatment of cholestasis, since budesonide in combination with UDCA stimulates activity of the Cl ^-^/HCO _3_^-^ exchanger AE2, thereby promoting bicarbonate-rich choleresis ^71^. Other interesting molecular targets comprise the membrane-located bile acid receptor TGR5, the xenobiotic receptor pregnane X receptor, or the vitamin D receptor. The reader is referred to recent reviews for a detailed overview on these pharmacological anticholestatic drug targets ^24^. Some of the hereditary cholestatic disorders are caused by mutations resulting in mistargeting of misfolded bile acid transporters to their intended subcellular location. Chemical chaperones have been shown to improve targeting of misfolded ATP8B1, MDR3, and BSEP transporters *in vitro* but also *in vivo* and may provide a pharmacological treatment option for specific hereditary mutations ^72– 75^.

## Summary and outlook

Recent understandings of the molecular mechanisms of bile formation and the enterohepatic circulation have revealed new molecular targets for treating cholestasis. Conceptually, the most promising drugs either stimulate bile flow as their main principle of action or decrease bile acid pool size. Both strategies decrease cholestatic injury in animal models and also appear to translate their observed effects into human clinical trials. However, from what we have learned from the clinical trials so far, there will still remain a substantial percentage of patients who will not completely respond to novel treatment regimes. From a teleological point of view, it therefore would make sense to combine drugs which are choleretic and target impaired bile flow with drugs that reduce bile acid accumulation and decrease bile acid pool size to maximize overall anticholestatic effects. Perhaps the prototypical compounds are the new FXR ligands, which appear to combine both effects, substantial suppression of bile acid synthesis and increasing bile acid-independent bile flow. Future strategies which combine the effects of the most powerful drugs to induce bicarbonate-rich choleresis, such as NorUDCA, with the most powerful drugs to suppress bile acid pool size, such as FGF19 mimetics or ASBT inhibitors, may therefore have real potential to heal cholestasis. Notably, several of these approaches also have profound anti-inflammatory and immunomodulatory actions, which may be instrumental in treating immune-mediated cholangiopathies. Surgical treatment strategies in severely cholestatic children with hereditary cholestatic defects also suggest that total biliary diversion might be a treatment option to avoid liver transplantation ^76^. However, surgery is complex and post-surgical complications can occur. Notably, some of these pharmacological approaches can be combined. As such, combination of ASBT inhibitors with FGF19 agonists may be a therapeutic way to pharmacologically mimic total biliary diversion and thus provide another rationale to combine new anticholestatic drugs to eventually heal cholestasis.

## Abbreviations

AE2, anion exchanger 2; AP, alkaline phosphatase; ASBT, apical sodium-dependent bile acid transporter; C4, 4-cholesten-3-one; CA, cholic acid; CDCA, chenodeoxycholic acid; CFTR, cystic fibrosis transmembrane conductance regulator; CYP3A4, cytochrome p450 3A4; CYP7A1, cholesterol 7-alpha hydroxylase; DCA, deoxycholic acid; FGF19, fibroblast growth factor 19; FXR, farnesoid X receptor; LCA, lithocholic acid; MDR3, multidrug resistance protein 3, MRP2-4, multidrug resistance-associated protein 2–4; NAFLD, non-alcoholic fatty liver disease; NTCP, sodium taurocholate cotransporting polypeptide; OCA, obeticholic acid; OSTα/β, organic solute transporter α/β; PBC, primary biliary cholangitis; PSC, primary sclerosing cholangitis; SHP, short heterodimer partner 1; TGR5, G-protein-coupled bile acid receptor 1; UDCA, ursodeoxycholic acid.

## References

[1] Hirschfield GM: Genetic determinants of cholestasis. *Clin Liver Dis.* 2013;17(2):147–59. 10.1016/j.cld.2012.12.002 23540495

[2] Monte MJ Marin JJ Antelo A: Bile acids: chemistry, physiology, and pathophysiology. *World J Gastroenterol.* 2009;15(7):804–16. 10.3748/wjg.15.804 19230041 PMC2653380

[3] Gomez-Ospina N Potter CJ Xiao R: Mutations in the nuclear bile acid receptor FXR cause progressive familial intrahepatic cholestasis. *Nat Commun.* 2016;7:10713. 10.1038/ncomms10713 26888176 PMC4759630

[4] Vaz FM Paulusma CC Huidekoper H: Sodium taurocholate cotransporting polypeptide (SLC10A1) deficiency: conjugated hypercholanemia without a clear clinical phenotype. *Hepatology.* 2015;61(1):260–7. 10.1002/hep.27240 24867799

[5] Poupon R Ping C Chrétien Y: Genetic factors of susceptibility and of severity in primary biliary cirrhosis. *J Hepatol.* 2008;49(6):1038–45. 10.1016/j.jhep.2008.07.027 18930330

[6] Sambrotta M Strautnieks S Papouli E: Mutations in *TJP2* cause progressive cholestatic liver disease. *Nat Genet.* 2014;46(4):326–8. 10.1038/ng.2918 24614073 PMC4061468

[7] Geier A Fickert P Trauner M: Mechanisms of disease: mechanisms and clinical implications of cholestasis in sepsis. *Nat Clin Pract Gastroenterol Hepatol.* 2006;3(10):574–85. 10.1038/ncpgasthep0602 17008927

[8] Russell DW: The enzymes, regulation, and genetics of bile acid synthesis. *Annu Rev Biochem.* 2003;72:137–74. 10.1146/annurev.biochem.72.121801.161712 12543708

[9] Wagner M Zollner G Trauner M: New molecular insights into the mechanisms of cholestasis. *J Hepatol.* 2009;51(3):565–80. 10.1016/j.jhep.2009.05.012 19595470

[10] Thomas C Pellicciari R Pruzanski M: Targeting bile-acid signalling for metabolic diseases. *Nat Rev Drug Discov.* 2008;7(8):678–93. 10.1038/nrd2619 18670431

[11] Boyer JL: Bile formation and secretion. *Compr Physiol.* 2013;3(3):1035–78. 10.1002/cphy.c120027 23897680 PMC4091928

[12] Beuers U Maroni L Elferink RO: The biliary HCO(3)(-) umbrella: experimental evidence revisited. *Curr Opin Gastroenterol.* 2012;28(3):253–7. 10.1097/MOG.0b013e328352aab2 22450897

[13] Trauner M Wagner M Fickert P: Molecular regulation of hepatobiliary transport systems: clinical implications for understanding and treating cholestasis. *J Clin Gastroenterol.* 2005;39(4 Suppl 2):S111–24. 10.1097/01.mcg.0000155551.37266.26 15758646

[14] Inagaki T Choi M Moschetta A: Fibroblast growth factor 15 functions as an enterohepatic signal to regulate bile acid homeostasis. *Cell Metab.* 2005;2(4):217–25. 10.1016/j.cmet.2005.09.001 16213224

[15] Pandak WM Li YC Chiang JY: Regulation of cholesterol 7 alpha-hydroxylase mRNA and transcriptional activity by taurocholate and cholesterol in the chronic biliary diverted rat. *J Biol Chem.* 1991;266(6):3416–21. 1995604

[16] Matsubara T Li F Gonzalez FJ: FXR signaling in the enterohepatic system. *Mol Cell Endocrinol.* 2013;368(1–2):17–29. 10.1016/j.mce.2012.05.004 22609541 PMC3491147

[17] Lefebvre P Cariou B Lien F: Role of bile acids and bile acid receptors in metabolic regulation. *Physiol Rev.* 2009;89(1):147–91. 10.1152/physrev.00010.2008 19126757

[18] Wang L Lee YK Bundman D: Redundant pathways for negative feedback regulation of bile acid production. *Dev Cell.* 2002;2(6):721–31. 10.1016/S1534-5807(02)00187-9 12062085

[19] Bookout AL Jeong Y Downes M: Anatomical profiling of nuclear receptor expression reveals a hierarchical transcriptional network. *Cell.* 2006;126(4):789–99. 10.1016/j.cell.2006.06.049 16923397 PMC6211849

[20] Moschetta A Bookout AL Mangelsdorf DJ: Prevention of cholesterol gallstone disease by FXR agonists in a mouse model. *Nat Med.* 2004;10(12):1352–8. 10.1038/nm1138 15558057

[21] Wang YD Chen WD Wang M: Farnesoid X receptor antagonizes nuclear factor kappaB in hepatic inflammatory response. *Hepatology.* 2008;48(5):1632–43. 10.1002/hep.22519 18972444 PMC3056574

[22] Wagner M Zollner G Trauner M: Nuclear bile acid receptor farnesoid X receptor meets nuclear factor-kappaB: new insights into hepatic inflammation. *Hepatology.* 2008;48(5):1383–6. 10.1002/hep.22668 18972560

[23] Zhang H Liu Y Bian Z: The critical role of myeloid-derived suppressor cells and FXR activation in immune-mediated liver injury. *J Autoimmun.* 2014;53:55–66. 10.1016/j.jaut.2014.02.010 24721598

[24] Beuers U Trauner M Jansen P: New paradigms in the treatment of hepatic cholestasis: from UDCA to FXR, PXR and beyond. *J Hepatol.* 2015;62(1 Suppl):S25–37. 10.1016/j.jhep.2015.02.023 25920087

[25] Lindor KD Gershwin ME Poupon R: Primary biliary cirrhosis. *Hepatology.* 2009;50(1):291–308. 10.1002/hep.22906 19554543

[26] Poropat G Giljaca V Stimac D: Bile acids for primary sclerosing cholangitis. *Cochrane Database Syst Rev.* 2011; (1):CD003626. 10.1002/14651858.CD003626.pub2 21249655 PMC7163275

[27] Yoon YB Hagey LR Hofmann AF: Effect of side-chain shortening on the physiologic properties of bile acids: hepatic transport and effect on biliary secretion of 23-nor-ursodeoxycholate in rodents. *Gastroenterology.* 1986;90(4):837–52. 3949115 10.1016/0016-5085(86)90859-0

[28] Fickert P Wagner M Marschall HU: 24- *nor*Ursodeoxycholic acid is superior to ursodeoxycholic acid in the treatment of sclerosing cholangitis in *Mdr2 (Abcb4)* knockout mice. *Gastroenterology.* 2006;130(2):465–81. 10.1053/j.gastro.2005.10.018 16472600

[29] Halilbasic E Fiorotto R Fickert P: Side chain structure determines unique physiologic and therapeutic properties of *nor*ursodeoxycholic acid in *Mdr2* ^-/-^ mice. *Hepatology.* 2009;49(6):1972–81. 10.1002/hep.22891 19475687 PMC3569724

[30] Moustafa T Fickert P Magnes C: Alterations in lipid metabolism mediate inflammation, fibrosis, and proliferation in a mouse model of chronic cholestatic liver injury. *Gastroenterology.* 2012;142(1):140–151.e12. 10.1053/j.gastro.2011.09.051 22001865

[31] Fickert P Pollheimer MJ Silbert D: Differential effects of *nor*UDCA and UDCA in obstructive cholestasis in mice. *J Hepatol.* 2013;58(6):1201–8. 10.1016/j.jhep.2013.01.026 23369794 PMC3650580

[32] Trauner M Halilbasic E Claudel T: Potential of *nor*-Ursodeoxycholic Acid in Cholestatic and Metabolic Disorders. *Dig Dis.* 2015;33(3):433–9. 10.1159/000371904 26045280

[33] Pellicciari R Fiorucci S Camaioni E: 6alpha-ethyl-chenodeoxycholic acid (6-ECDCA), a potent and selective FXR agonist endowed with anticholestatic activity. *J Med Chem.* 2002;45(17):3569–72. 10.1021/jm025529g 12166927

[34] Fiorucci S Clerici C Antonelli E: Protective effects of 6-ethyl chenodeoxycholic acid, a farnesoid X receptor ligand, in estrogen-induced cholestasis. *J Pharmacol Exp Ther.* 2005;313(2):604–12. 10.1124/jpet.104.079665 15644430

[35] Baghdasaryan A Claudel T Gumhold J: Dual farnesoid X receptor/TGR5 agonist INT-767 reduces liver injury in the *Mdr*2 ^-/-^ *(Abcb4 ^-/-^)* mouse cholangiopathy model by promoting biliary HCO ^-^ _3_ output. *Hepatology.* 2011;54(4):1303–12. 10.1002/hep.24537 22006858 PMC3744065

[36] Liu Y Binz J Numerick MJ: Hepatoprotection by the farnesoid X receptor agonist GW4064 in rat models of intra- and extrahepatic cholestasis. *J Clin Invest.* 2003;112(11):1678–87. 10.1172/JCI18945 14623915 PMC281645

[37] Modica S Petruzzelli M Bellafante E: Selective activation of nuclear bile acid receptor FXR in the intestine protects mice against cholestasis. *Gastroenterology.* 2012;142(2):355–65.e1–4. 10.1053/j.gastro.2011.10.028 22057115

[38] Fang S Suh JM Reilly SM: Intestinal FXR agonism promotes adipose tissue browning and reduces obesity and insulin resistance. *Nat Med.* 2015;21(2):159–65. 10.1038/nm.3760 25559344 PMC4320010

[39] Hirschfield GM Mason A Luketic V: Efficacy of obeticholic acid in patients with primary biliary cirrhosis and inadequate response to ursodeoxycholic acid. *Gastroenterology.* 2015;148(4):751–61.e8. 10.1053/j.gastro.2014.12.005 25500425

[40] Neuschwander-Tetri BA Loomba R Sanyal AJ: Farnesoid X nuclear receptor ligand obeticholic acid for non-cirrhotic, non-alcoholic steatohepatitis (FLINT): a multicentre, randomised, placebo-controlled trial. *Lancet.* 2015;385(9972):956–65. 10.1016/S0140-6736(14)61933-4 25468160 PMC4447192

[41] Neuschwander-Tetri BA: Targeting the FXR nuclear receptor to treat liver disease. *Gastroenterology.* 2015;148(4):704–6. 10.1053/j.gastro.2015.02.037 25724453

[42] Nevens F Andreone P Mazzella G: O168 the first primary biliary cirrhosis (PBC) phase 3 trial in two decades – an international study of the FXR agonist obeticholic acid in PBC patients. *J Hepatol.* 2014;60(1):S525–S526. 10.1016/S0168-8278(14)61463-X

[43] Trauner M Nevens F Andreone P: Sustained improvement in the markers of cholestasis in an open label long term safety extension study of obeticholic acid in primary biliary cirrhosis patients. *Hepatology.* 2015;62:511A (Abstract) Reference Source

[44] Wunsch E Milkiewicz M Wasik U: Expression of hepatic Fibroblast Growth Factor 19 is enhanced in Primary Biliary Cirrhosis and correlates with severity of the disease. *Sci Rep.* 2015;5: 13462. 10.1038/srep13462 26293907 PMC4544021

[45] Zweers SJ Booij KA Komuta M: The human gallbladder secretes fibroblast growth factor 19 into bile: towards defining the role of fibroblast growth factor 19 in the enterobiliary tract. *Hepatology.* 2012;55(2):575–83. 10.1002/hep.24702 21953282

[46] Luo J Ko B Elliott M: A nontumorigenic variant of FGF19 treats cholestatic liver diseases. *Sci Transl Med.* 2014;6(247):247ra100. 10.1126/scitranslmed.3009098 25080475

[47] Nicholes K Guillet S Tomlinson E: A mouse model of hepatocellular carcinoma: ectopic expression of fibroblast growth factor 19 in skeletal muscle of transgenic mice. *Am J Pathol.* 2002;160(6):2295–307. 10.1016/S0002-9440(10)61177-7 12057932 PMC1850847

[48] Sawey ET Chanrion M Cai C: Identification of a therapeutic strategy targeting amplified *FGF19* in liver cancer by Oncogenomic screening. *Cancer Cell.* 2011;19(3):347–58. 10.1016/j.ccr.2011.01.040 21397858 PMC3061399

[49] Zhou M Learned RM Rossi SJ: Engineered fibroblast growth factor 19 reduces liver injury and resolves sclerosing cholangitis in *Mdr2*-deficient mice. *Hepatology.* 2016;63(3):914–29. 10.1002/hep.28257 26418580 PMC5063176

[50] Mayo MJ Roberts SK Arnold H: NGM282, A Novel Variant of FGF-19, Demonstrates Biologic Activity in Primary Biliary Cirrhosis Patients with an Incomplete Response to Ursodeoxycholic Acid: Results of a Phase 2 Multicenter, Randomized, Double Blinded, Placebo Controlled Trial. *Hepatology.* 2015;62(Suppl. 1):263A (Abstract) Reference Source

[51] Kir S Beddow SA Samuel VT: FGF19 as a postprandial, insulin-independent activator of hepatic protein and glycogen synthesis. *Science.* 2011;331(6024):1621–4. 10.1126/science.1198363 21436455 PMC3076083

[52] Walters JR Tasleem AM Omer OS: A new mechanism for bile acid diarrhea: defective feedback inhibition of bile acid biosynthesis. *Clin Gastroenterol Hepatol.* 2009;7(11):1189–94. 10.1016/j.cgh.2009.04.024 19426836

[53] Walters JR: Bile acid diarrhoea and FGF19: new views on diagnosis, pathogenesis and therapy. *Nat Rev Gastroenterol Hepatol.* 2014;11(7):426–34. 10.1038/nrgastro.2014.32 24662279

[54] Owen BM Mangelsdorf DJ Kliewer SA: Tissue-specific actions of the metabolic hormones FGF15/19 and FGF21. *Trends Endocrinol Metab.* 2015;26(1):22–9. 10.1016/j.tem.2014.10.002 25476453 PMC4277911

[55] Rysz J Gluba-Brzózka A Mikhailidis DP: Fibroblast growth factor 19-targeted therapies for the treatment of metabolic disease. *Expert Opin Investig Drugs.* 2015;24(5):603–10. 10.1517/13543784.2015.1006357 25604607

[56] Dawson PA Haywood J Craddock AL: Targeted deletion of the ileal bile acid transporter eliminates enterohepatic cycling of bile acids in mice. *J Biol Chem.* 2003;278(36):33920–7. 10.1074/jbc.M306370200 12819193

[57] Lundåsen T Andersson EM Snaith M: Inhibition of intestinal bile acid transporter Slc10a2 improves triglyceride metabolism and normalizes elevated plasma glucose levels in mice. *PLoS One.* 2012;7(5):e37787. 10.1371/journal.pone.0037787 22662222 PMC3360597

[58] Trivedi PJ Ward S: Altered bile acid pool using IBAT inhibitors for constipation: a potentially increased risk of malignancy. *Am J Gastroenterol.* 2012;107(1):140; author reply 140–1. 10.1038/ajg.2011.378 22218036

[59] Raufman JP Dawson PA Rao A: *Slc10a2*-null mice uncover colon cancer-promoting actions of endogenous fecal bile acids. *Carcinogenesis.* 2015;36(10):1193–200. 10.1093/carcin/bgv107 26210740 PMC4612337

[60] Miethke AG Zhang W Simmons J: Pharmacological inhibition of apical sodium-dependent bile acid transporter changes bile composition and blocks progression of sclerosing cholangitis in multidrug resistance 2 knockout mice. *Hepatology.* 2016;63(2):512–23. 10.1002/hep.27973 26172874 PMC4713368

[61] Baghdasaryan A Fuchs CD Österreicher CH: Inhibition of intestinal bile acid absorption improves cholestatic liver and bile duct injury in a mouse model of sclerosing cholangitis. *J Hepatol.* 2016;64(3):674–81. 10.1016/j.jhep.2015.10.024 26529078

[62] Marschall HU Gillberg P Graffner H: The ileal bile acid transporter inhibitor A4250 modulates bile acid synthesis and decreases serum bile acids. *Hepatology.* 2015;62:612A (Abstract).

[63] Jacobson TA Armani A McKenney JM: Safety considerations with gastrointestinally active lipid-lowering drugs. *Am J Cardiol.* 2007;99(6A):47C–55C. 10.1016/j.amjcard.2006.11.022 17368279

[64] Harach T Pols TW Nomura M: TGR5 potentiates GLP-1 secretion in response to anionic exchange resins. *Sci Rep.* 2012;2:430. 10.1038/srep00430 22666533 PMC3362799

[65] Inagaki T Moschetta A Lee Y: Regulation of antibacterial defense in the small intestine by the nuclear bile acid receptor. *Proc Natl Acad Sci U S A.* 2006;103(10):3920–5. 10.1073/pnas.0509592103 16473946 PMC1450165

[66] Fouts DE Torralba M Nelson KE: Bacterial translocation and changes in the intestinal microbiome in mouse models of liver disease. *J Hepatol.* 2012;56(6):1283–92. 10.1016/j.jhep.2012.01.019 22326468 PMC3357486

[67] Honda A Ikegami T Nakamuta M: Anticholestatic effects of bezafibrate in patients with primary biliary cirrhosis treated with ursodeoxycholic acid. *Hepatology.* 2013;57(5):1931–41. 10.1002/hep.26018 22911624

[68] Kok T Bloks VW Wolters H: Peroxisome proliferator-activated receptor alpha (PPARalpha)-mediated regulation of multidrug resistance 2 (Mdr2) expression and function in mice. *Biochem J.* 2003;369(Pt 3):539–47. 10.1042/BJ20020981 12381268 PMC1223107

[69] Shoda J Inada Y Tsuji A: Bezafibrate stimulates canalicular localization of NBD-labeled PC in HepG _2_ cells by PPARalpha-mediated redistribution of ABCB _4_. *J Lipid Res.* 2004;45(10):1813–25. 10.1194/jlr.M400132-JLR200 15258199

[70] Wagner M Zollner G Trauner M: Nuclear receptors in liver disease. *Hepatology.* 2011;53(3):1023–34. 10.1002/hep.24148 21319202

[71] Arenas F Hervias I Uriz M: Combination of ursodeoxycholic acid and glucocorticoids upregulates the AE2 alternate promoter in human liver cells. *J Clin Invest.* 2008;118(2):695–709. 18188457 10.1172/JCI33156 PMC2176189

[72] Gonzales E Grosse B Schuller B: Targeted pharmacotherapy in progressive familial intrahepatic cholestasis type 2: Evidence for improvement of cholestasis with 4-phenylbutyrate. *Hepatology.* 2015;62(2):558–66. 10.1002/hep.27767 25716872

[73] Gonzales E Grosse B Cassio D: Successful mutation-specific chaperone therapy with 4-phenylbutyrate in a child with progressive familial intrahepatic cholestasis type 2. *J Hepatol.* 2012;57(3):695–8. 10.1016/j.jhep.2012.04.017 22609309

[74] Gautherot J Durand-Schneider A Delautier D: Effects of cellular, chemical, and pharmacological chaperones on the rescue of a trafficking-defective mutant of the ATP-binding cassette transporter proteins ABCB _1_/ABCB _4_. *J Biol Chem.* 2012;287(7):5070–8. 10.1074/jbc.M111.275438 22184139 PMC3281624

[75] van der Velden LM Lieke JM Stapelbroek JM: Folding defects in P-type ATP 8B1 associated with hereditary cholestasis are ameliorated by 4-phenylbutyrate. *Hepatology.* 2010;51(1):286–96. 10.1002/hep.23268 19918981

[76] van der Woerd WL Kokke FT van der Zee DC: Total biliary diversion as a treatment option for patients with progressive familial intrahepatic cholestasis and Alagille syndrome. *J Pediatr Surg.* 2015;50(11):1846–9. 10.1016/j.jpedsurg.2015.07.016 26319776

